# The use of hormonal therapy with radiotherapy for prostate cancer: analysis of prospective randomised trials

**DOI:** 10.1038/sj.bjc.6601625

**Published:** 2004-03-02

**Authors:** A R Gottschalk, M Roach

**Affiliations:** 1 Department of Radiation Oncology and the Comprehensive Cancer Center, University of California, San Francisco, 1600 Divisadero St., H-1031, San Francisco, CA 94115, USA

**Keywords:** prostate cancer, hormonal therapy, radiation, whole-pelvic radiation, randomised trials

## Abstract

In 1901, Wilhelm Conrad Röntgen won the Nobel prize in Physics for his discovery of the Röntgen rays or, as he himself called them, X-rays. In 1966, Dr Charles Brenton Higgins won the Nobel Prize in Medicine for his breakthroughs concerning hormonal treatment of prostatic cancer. After 31 years, in 1997, the first prospective randomised trials of the combination of hormonal therapy and radiation therapy were published, showing increased survival when compared to radiation therapy alone for patients with prostate cancer. Since 1997, many investigators have published trials combining hormonal and radiation therapy for prostate cancer. This minireview will address the largest and most influential of these trials, and attempt to guide physicians in selecting the appropriate patients for this combined approach.

Patients with prostate cancer are usually divided into risk groups in order to determine their chance of recurrence and appropriate therapy. Although there are many methods to define the risk groups, they all separate patients into low-risk, intermediate-risk and high-risk categories. Recurrences after therapy can be defined as local (prostate only), regional (lymph nodes) or distant (bone metastasis).

Low-risk patients do extremely well with treatment and have a low risk of a recurrence locally, regionally or distantly. It is generally accepted that these patients do not benefit from the addition of hormonal therapy, although hormonal therapy in low-risk patients has not been formally tested. Intermediate-risk patients have a greater potential for local-regional recurrences and distant recurrences. High-risk patients confront a significant risk of distant recurrence. While local-regional failure can occur in high-risk patients, distant disease is the main problem for these patients.

In this article, we will discuss prospective randomised trials demonstrating that intermediate-risk patients benefit from short-term androgen deprivation and high-risk patients benefit from long-term androgen deprivation (LTAD). These observations suggest that short-term androgen deprivation reduces the risk of a local-regional recurrence, while LTAD is effective in treating systemic disease.

## EORTC

As published in the New England Journal of Medicine in 1997 (Bolla *et al*, 1997), the EORTC reported a study of improved survival in patients with locally advanced prostate cancer treated with radiotherapy and goserlin. Patients eligible for this trial were T1–2 WHO grade 3, or T3–4 any grade. In all, 415 patients were randomised to either radiotherapy alone (50 Gy to the whole pelvis and a prostate boost to 70 Gy) or the same radiotherapy and 3.6 mg goserelin s.c. every 4 weeks, starting on the first day of radiotherapy and continuing for 3 years. Patients in the latter group also received cyproterone acetate (150 mg day^−1^ p.o.) during the first month of treatment to inhibit the transient rise in testosterone associated with the administration of goserelin.

In 1997, the median follow-up was 45 months. Kaplan–Meier estimates of the disease-free survival were 85 *vs* 48% (*P*<0.001) in favour of the combined-treatment group. Most impressive was the increase in overall survival: 79 *vs* 62% (*P*=0.001). The EORTC trial was updated in 2002 (Bolla *et al*, 2002) with a median follow-up of 66 months. The 2002 update showed that the difference in survival between the two groups was maintained over time. The disease-free survival was 74 *vs* 40% (*P*=0.0001) and the overall survival was 78 *vs* 62% (*P*=0.0002), in favour of the combined-modality arm.

For the EORTC trial, hormonal therapy was initiated on the first day of radiation therapy and continued for 3 years. The concurrent administration of hormonal therapy with radiation therapy may allow for a synergistic effect. As the radiotherapy was delivered to both the prostate and regional lymph nodes, the benefit of combined hormonal-radiation therapy may have been due to treatment of either of these areas. Investigation of the importance of lymph node radiation *vs* prostate radiation was the subject of another trial (RTOG 94-13), discussed later in this review.

The administration of adjuvant hormonal therapy may have a separate biologic effect from the concurrent administration of hormonal therapy with radiation therapy. Patients eligible for the EORTC trial had either high-grade tumour or cT3/cT4 tumour of any grade. Multivariate analysis revealed that disease-free survival was associated with an increased relative risk of 1.84 for WHO grade 3 tumours (Bolla *et al*, 2002). The benefit seen with the long-term use of adjuvant hormonal therapy may be due to the large number of high-grade tumours in the trial. Tumour grade predicts for metastatic disease, and the use of long-term adjuvant hormonal therapy may allow for treatment of possible micro-metastatic disease.

## RTOG 85-31

RTOG 85-31 was a phase III randomised trial of radiation therapy alone *vs* radiation therapy, followed by adjuvant goserelin for life. The patient eligibility for this trial was lymph node positive, cT3 or pT3. In both arms, the radiation therapy was 44–46 Gy to the whole pelvis, followed by a boost to the prostate to a total dose of 65–70 Gy. Adjuvant goserelin was started during the last week of radiation therapy and continued indefinitely or until signs of disease progression.

Initial analysis of the data from RTOG 85-31 showed that the adjuvant goserelin arm had a benefit in local control (84 *vs* 71%, *P*<0.0001), a decrease in distant metastases (17 *vs* 30%, *P*<0.001) and an increase in disease-free survival (60 *vs* 44%, *P*<0.0001), but no difference in overall survival for all patients. However, in patients with centrally reviewed tumours with a Gleason score (GS) of 8–10, the difference in 5-year overall survival was 66 *vs* 55%, favouring the adjuvant goserelin arm (*P*=0.03) (Pilepich *et al*, 1997). With additional follow-up (median 10 years), adjuvant goserelin showed a benefit for all patients in the study. Adjuvant hormonal therapy demonstrated increased local control (77 *vs* 61%), decreased distant metastases (25 *vs* 39%) and improved overall survival (53 *vs* 38%) (Pilepich *et al*, 2003).

The fact that radiation and hormonal therapies overlap for only 1 week suggests that the improvement in overall survival seen in this study is not due to a synergistic effect within the radiation field. Instead, an additive effect of local treatment with radiation, and systemic therapy with androgen deprivation, is the most likely explanation of the observed benefit.

Similar to the EORTC trial, patients with high-grade (Gleason 8–10) tumours benefited most from long-term hormonal therapy. This supports the idea that patients with high-grade tumours are most likely to develop distant metastases and die from prostate cancer, and that long-term hormonal therapy must have a significant systemic effect in order to decrease the incidence of distant metastases and improve the overall survival.

## RTOG 86-10

RTOG 86-10 was a phase III randomised trial of external-beam radiation therapy +/− combined androgen blockade (CAB) for locally advanced prostate cancer patients. The eligible patients had bulky tumours (palpable 25 cm^2^ or more), clinical stage T2–4. The standard arm received external-beam radiation therapy, 44–46 Gy to the prostate, SV and pelvic lymph nodes, followed by a prostate boost to 65–70 Gy. The experimental arm was the same radiotherapy, plus goserelin and flutamide. Goserelin was given 3.6 mg s.c. every 4 weeks and flutamide 250 mg p.o. three times a day for 2 months before and 2 months during radiation therapy.

The initial RTOG 86-10 report was published in 1995 (Pilepich *et al*, 1995), with a median follow-up of 4.5 years. The publication documented a cumulative incidence of local progression at 5 years of 71% with radiation alone, and 45% with radiation plus short-term CAB (*P*<0.001). Progression-free survival was also increased (*P*<0.001) in the hormonal therapy arm: 30 *vs* 15% with radiation alone. There was no difference in overall survival.

In 2001, a follow-up of this study was published (Pilepich *et al*, 2001), with a median follow-up of 6.7 years for all patients, and 8.6 years for living patients. The arm containing CAB was associated with improved local control (42 *vs* 30%, *P*=0.016), reduced incidence of bone metastases (34 *vs* 45%, *P*=0.04), increased disease-free survival (33 *vs* 21%, *P*=0.004), but no overall survival advantage to the group as a whole. However, on subset analysis of patients with a GS 2–6, there was an overall survival advantage (70 *vs* 52%, *P*=0.015).

The group of patients that benefited the most in RTOG 86-10 were patients with bulky disease and a GS of 2–6. Patients with higher-grade tumours did not benefit, probably because they require long-term hormonal therapy, similar to the protocol used in the EORTC study and RTOG 85-31. Today, with widespread use of PSA screening, we rarely see patients present with palpable disease measuring 25 cm^2^. Instead, modern day bulk disease may represent any patient with significant palpable disease on digital rectal examination (clinical stage T2b, T2c, T3a or T3b). These patients will most likely benefit from the use of hormonal therapy in combination with radiotherapy.

The percentage of positive biopsies may also be used to determine who may benefit from short-term hormonal therapy. In a concept popularised by D'Amico, percent positive biopsies can be an independent predictor of biochemical failure. Patients with otherwise low-risk features may behave more like intermediate-risk patients if >50% of the biopsies are positive for cancer (D'Amico *et al*, 2001; D'Amico *et al*, 2002). These patients with >50% positive biopsies may represent ‘bulky disease’ in today's PSA era and, therefore, benefit from short-term hormonal therapy.

It is important to note that the hormonal therapy in this trial was given as neoadjuvant and concurrent therapy, and that whole-pelvic radiotherapy was also used. This allows for a synergistic effect of the hormonal therapy and radiotherapy on the lymph nodes and/or prostate. The 4-month duration of hormonal therapy might also have a systemic effect, as there was a decrease in bone metastasis in the hormonal therapy group. Whether there is a difference between short-term (4 months) and long-term (2–3 years) hormonal therapy was the question addressed by RTOG 92-02.

## RTOG 92-02

RTOG 92-02 was a prospective randomised trial testing the duration of androgen suppression with external beam radiation in patients with locally advanced prostate cancer (Table 1

**Table 1:** Summary of five prospective randomised trials involving hormonal and radiation therapy in patients with prostate cancer

	**EORTC**	**RTOG 85-31**	**RTOG 86-10**	**RTOG 92-02**	**RTOG 94-13**
*n*	415	977	471	1554	1323
Eligibility	T1–2, WHO grade 3	cT3, pT3 or N1	T2–4 with palpable tumour 25 cm^2^	T2c–4 PSA<150	PSA<100
	T3–4, any grade				Risk of LN involvement>15%
Median follow-up	66 months	7.3 years (all)	6.7 years (all)	4.8 years	5 years
		10 years (alive)	8.6 years (alive)		
Arms	XRT *vs* XRT+goserelin starting day 1 for 3 years	XRT *vs* XRT+adj goserelin for life	XRT *vs* XRT+CAB 2 months before 2 months during	XRT+CAB 2 months before+2 months during *vs* same+2 years adjuvant goserelin	WP+N&CHT *vs* PO+N&CHT *vs* WP+AHT *vs* PO+AHT
Whole pelvis	Yes	Yes	Yes	Yes	Yes
					Arms 1 and 3
Dose	50 Gy wp	44–46 Gy wp	44–46 Gy wp	44–50 Gy wp	50.4 wp
	70 Gy prostate	65–70 Gy p	65–70 Gy p	65–70 Gy p	70.2 po
Local-regional control	72 *vs* 97%	61 *vs* 77%	42 *vs* 30%	94 *vs* 87%	NR
Distant mets	56 *vs* 22%	39 *vs* 25%	34 *vs* 45%	11 *vs* 17%	NR
Disease-free survival	40 *vs* 74%	NR	33 *vs* 21 %	54 *vs* 34%	60 *vs* 44 *vs* 49 *vs* 50%
Overall survival	62 *vs* 78% all patients	38 *vs* 53% all patients	70 *vs* 52% for GS 2–6	80 *vs* 69% for GS 8–10	NR
*P*-value	0.0002	<0.0043	0.015	0.007	

CAB=combined androgen blockade; GS=Gleason score; AHT=adjuvant hormone therapy; NR=not reported.

). Eligibility included T2C–4 with PSA <150 ng ml^−1^. All patients received 4 months of goserelin and flutamide, 2 months before and 2 months during radiation, and then were randomised to either no further therapy or 24 months of additional goserelin alone. Radiation doses were 44–50 Gy to the whole pelvis and 65–70 Gy to the prostate. This trial entered 1554 patients, and reported results from a median follow-up of 4.8 years.

Initially published in abstract form (Hanks *et al*, 2000), the group with LTAD showed a significant improvement in disease-free survival (54 *vs* 34%, *P*=0.0001), clinical local progression (6.2 *vs* 13%, *P*=0.0001), freedom from distant metastasis (11 *vs* 17%, *P*=0.001) and ASTRO-defined biochemical control (21 *vs* 46%, *P*=0.0001). There was no difference in the 5-year overall survival between the two arms (78 *vs* 79%).

In a subset analysis of T3, T4 and T2 with GS8–10 (the same criteria used for Bolla *et al*, 1997), LTAD showed an advantage in disease-free survival (90 *vs* 86%, *P*=0.03), but no difference in overall survival. In the subset analysis of all GS8–10 patients (the group that benefited most from hormonal therapy in RTOG 85-31), the 5-year overall survival (80 *vs* 69%, *P*=0.02) and disease-free survival (90 *vs* 78%, *P*=0.007) were in favour of the LTAD group.

The data from RTOG 92-02 support the findings from the EORTC trial and RTOG 85-31. Patients with high-grade (GS8–10) tumours benefit most from LTAD. Data from all the three trials show an increase in overall survival when patents with high-grade tumours are treated with long-term hormonal therapy. The exact duration of hormonal therapy is still in question. RTOG 92-02, EORTC and RTOG 85-31 used 2 years, 3 years and unlimited years, respectively. Since there is no evidence to support that greater than 2 years of LTAD is better than 2 years, we suggest that patients with high-grade tumours be treated with 2 years of adjuvant hormonal therapy.

## RTOG 94-13

The eligibility for RTOG 94-13 was limited to clinically localised adenocarcinoma of the prostate, with elevated PSA of less than or equal to 100. Patients were stratified by T-stage, PSA and GS, and were required to have an estimated risk of lymph node involvement >15%, based on the equation: risk of positive nodes=(2/3) PSA+((GS)−6) × 10) (Partin *et al*, 1993; Roach, 1993).

In RTOG 94-13, CAB was used and consisted of flutamide 250 mg p.o. t.i.d. and either leuprolide or goserelin acetate, administered 2 months before and 2 months during radiotherapy (N&CHT), or 4 months following the completion of radiotherapy (adjuvant hormone therapy (AHT)). Patients were randomised to one of four arms: arm 1 received whole-pelvic radiotherapy and N&CHT, arm 2 received prostate only radiotherapy and N&CHT, arm 3 received whole-pelvic radiotherapy plus AHT, and arm 4 received prostate only radiotherapy plus AHT.

With a median follow-up for all patients of 59.3 months, the study showed that patients treated with whole-pelvic radiotherapy experienced a 4-year progression-free survival of 56% compared to 40% for prostate only (*P*=0.014). Comparing all the four arms, there was a progression-free survival advantage for arm 1 (61%) compared to the other three arms (45, 49 and 47%, respectively; *P*=0.008) (Roach *et al*, 2003). Longer follow-up is needed to address the issue of disease-specific and overall survival. However, even without survival data, the findings still reveal the value of whole-pelvic radiotherapy.

**Figure 1:**
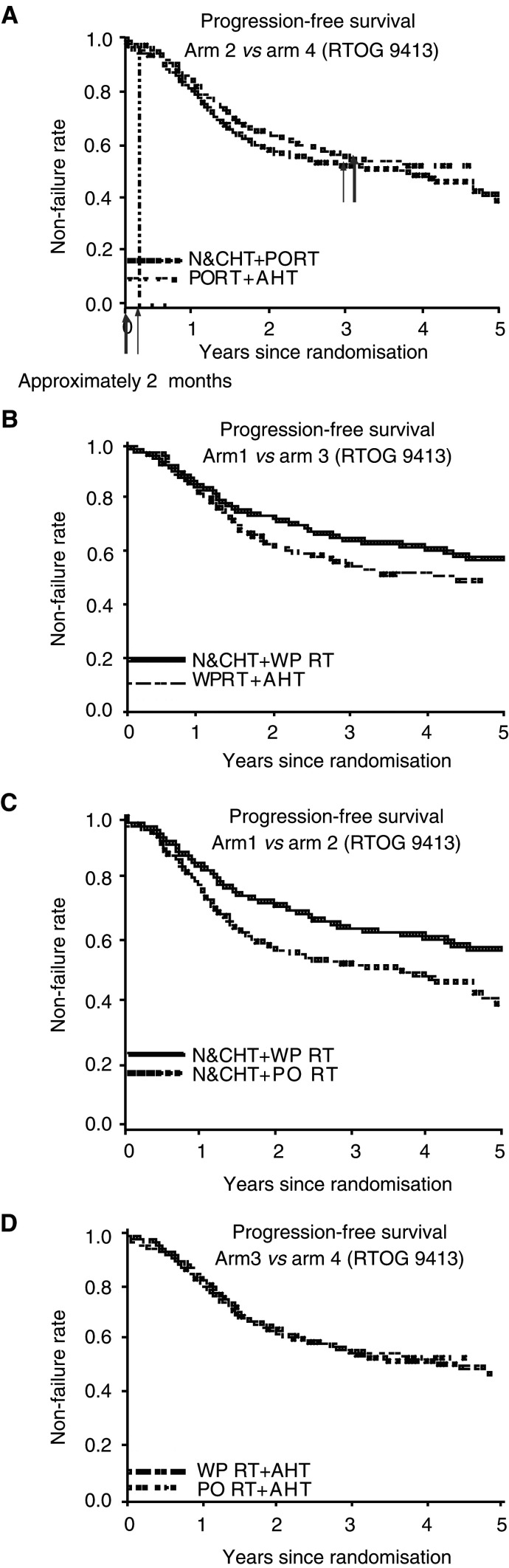
Progression-free survival for the four arms of RTOG 94-13. (**A**) PO+N&CHT *vs* PO+AHT. (**B**) WP+N&CHT *vs* WP+AHT. (**C**) WP+N&CHT *vs* PO+N&CHT. (**D**) WP+AHT *vs* PO+AHT (modified with permission from Roach *et al*, 2003).

Before discussing the individual arms in more detail, it is important to mention a quirk of the study design. Patients treated in arms 1 and 2 are subject to a worse outcome relative to arms 3 and 4, because of the timing of hormonal therapy. The time of failure was measured from the time of randomisation. Patients in arms 1 and 2 were treated with N&CHT and received CAB therapy for months 1 and 2, and concurrent hormone therapy plus radiation in months 3 and 4, so that the total duration of treatment was 4 months. In contrast, patients treated in arms 3 and 4 received 2 months of radiation followed by 4 months of AHT, so that their total duration of treatment was 6 months. Thus, when measured from the time of randomisation, the patients receiving AHT were likelier to be disease-free at least 2 months later, because of the time at which hormone therapy was discontinued. Figure 1A

demonstrates this effect by comparing arms 2 and 4 (both prostate-only arms) separately from the other curves. These curves are consistent with a time-to-failure bias in favour of AHT compared to N&CHT, of approximately 2 months. This curve suggests that, whether hormonal therapy is given in an N&CHT fashion or as an AHT after EBRT, there is no evidence of a difference in biologic interactions if only the prostate is irradiated.

Next, it is instructive to compare the two whole-pelvic arms, the one receiving N&CHT (arm 1) and the one receiving AHT (arm 3). Here, as with the prostate-only arms, there is a bias in favour of the AHT arm in disease-free survival due to the timing of hormone therapy. Despite this bias, the curves overlap in the early part of the curve, but separate with longer follow-up (Figure 1B). This suggests that there is a greater biologic interaction when hormonal therapy is given before and during, rather than after, whole-pelvic radiotherapy.

Additional evidence for the benefits of whole-pelvic radiotherapy is seen in the comparison of arms 1 and 2, both using N&CHT (Figure 1C). Arm 1 included the whole pelvis, while arm 2 included only prostate. This comparison eliminates the biases associated with AHT and demonstrates a very large effect of whole-pelvic treatment. This was further supported by comparing arms 3 and 4, both of which used AHT (arm 3 including whole-pelvic treatment and arm 4 prostate-only radiotherapy) (Figure 1D). This curve suggests that there is no evidence of benefit to whole-pelvic radiotherapy if given with AHT. This very important observation suggests that the location of the biologic interaction is in the pelvic lymph nodes. The data from RTOG 94-13 clearly demonstrate that hormonal therapy given short term before and during radiotherapy enhances the biologic effect of whole-pelvic radiation treatment.

## RTOG META-ANALYSIS

A meta-analysis of RTOG 7506, 7706, 8531 and 8610 confirms the above observations of the advantage of hormonal therapy with radiation therapy. A total of 2742 men with prostate cancer were separated into four risk groups that are predictive of disease-free survival (Roach *et al*, 2000a). Group 1 patients are T1–2 and GS2–6; group 2 are T3 GS2–6 or T1–2 GS7; group 3 are T3 GS7 or T1–2 GS8–10; group 4 are T3 GS8–10. All patients were treated in the pre-PSA era; therefore, PSA was not used to define the risk groups. Patients in risk group 2 (either GS2–6 with bulky tumours, or GS7 with organ-confined disease) benefited from 4 months of flutamide and goserlin. Patients in risk groups 3 and 4 (T3 GS7 or GS8–10) benefited from long-term hormonal therapy (Roach *et al*, 2000b).

## FINAL RECOMMENDATIONS

Currently, the use of hormonal therapy falls into two categories: short-term and long-term hormonal therapy. The short-term category involves hormonal therapy 2 months before and 2 months during radiation. The long-term category involves short-term hormonal therapy plus 2 years of adjuvant hormonal therapy. Patients who should be considered for short-term hormonal therapy include patients with a risk of lymph node involvement >15%, or patients with bulky disease defined by either a large palpable mass in the prostate or >50% positive biopsies or T3 disease. It is important to remember that the benefit of short-term hormonal therapy is only seen when given with whole-pelvic radiation therapy. The patients who should be considered for long-term hormonal therapy are those with either GS8–10 tumours or possibly T3 GS7.

## FUTURE DIRECTIONS

There are a couple of current RTOG trials that are continuing to investigate the role of hormonal therapy for patients with prostate cancer. For intermediate-risk patients, there is RTOG 99-10, a phase III randomised trial comparing standard treatment (8 weeks neo-adjuvant CAB and 8 weeks concurrent CAB/XRT), with the experimental arm of 28 weeks neo-adjuvant CAB and 8 weeks concurrent CAB/XRT. The radiotherapy is identical in both arms. Whole-pelvic radiation is used for those patients with a risk of lymph node involvement >15%. RTOG 99-10 will address if longer neo-adjuvant CAB is beneficial in intermediate-risk patients.

For high-risk patients, there is RTOG 99-02. The standard arm in this trial is 2 months neo-adjuvant CAB, 2 months concurrent CAB/XRT plus 2 years of adjuvant LHRH agonist. The experimental arm involves the same therapy, plus adjuvant chemotherapy consisting of Emcyt, Toxol and Etoposide. This trial is addressing the question of whether additional chemotherapy can reduce the incidence of distant metastasis, which might improve survival in this high-risk population.

Much progress has been made in the treatment of prostate cancer over the past 6 years. With subsequent follow-up of past trials and completion of current trials, we will increase our knowledge of how to optimally treat prostate cancer patients with the combination of hormonal therapy and radiotherapy.
